# Total Body Irradiation Mitigates Inflammation and Extends the Therapeutic Time Window for Anti-Ricin Antibody Treatment against Pulmonary Ricinosis in Mice

**DOI:** 10.3390/toxins9090278

**Published:** 2017-09-11

**Authors:** Yoav Gal, Anita Sapoznikov, Reut Falach, Sharon Ehrlich, Moshe Aftalion, Chanoch Kronman, Tamar Sabo

**Affiliations:** Department of Biochemistry and Molecular Genetics, Israel Institute for Biological Research, Ness-Ziona 76100, Israel; yoavg@iibr.gov.il (Y.G.); anitas@iibr.gov.il (A.S.); reutf@iibr.gov.il (R.F.); sharone@iibr.gov.il (S.E.); moshea@iibr.gov.il (M.A.); tamars@iibr.gov.il (T.S.)

**Keywords:** ricin, total body irradiation, leukopenia, neutropenia, inflammation

## Abstract

Ricin, a highly toxic plant-derived toxin, is considered a potential weapon in biowarfare and bioterrorism due to its pronounced toxicity, high availability, and ease of preparation. Pulmonary exposure to ricin results in the generation of an acute edematous inflammation followed by respiratory insufficiency and death. Massive neutrophil recruitment to the lungs may contribute significantly to ricin-mediated morbidity. In this study, total body irradiation (TBI) served as a non-pharmacological tool to decrease the potential neutrophil-induced lung injury. TBI significantly postponed the time to death of intranasally ricin-intoxicated mice, given that leukopenia remained stable following intoxication. This increase in time to death coincided with a significant reduction in pro-inflammatory marker levels, and led to marked extension of the therapeutic time window for anti-ricin antibody treatment.

## 1. Introduction

Ricin, a type II ribosome inactivating protein (RIP), is a toxin derived from the seeds of *Ricinus communis* (the castor oil plant). The holotoxin consists of two polypeptide chains, ricin toxin B (RTB) and ricin toxin A (RTA), linked by a single disulfide bond. RTB binds to galactose residues on the surface of cells and mediates the toxin’s cellular internalization, whereas RTA possesses the catalytic activity of ricin [1].

Due to the high availability of the toxin and the relative ease of its production, ricin is considered a biological threat agent [2]. The toxicity of ricin depends on the route of exposure, the inhalational route being considered most fatal [3]. Pulmonary ricinosis comprises two pathological processes, ribosomal depurination and pulmonary inflammation, occurring at the molecular and cellular/tissue levels, respectively.

The RTA-dependent depurination is due to RNA N-glycosidase activity which cleaves a specific adenine residue located within the 28S rRNA of the mammalian 60S ribosome subunit [4,5,6,7]. This site-specific depurination event prevents binding of elongation factor-2 to the ribosome, thereby causing translational arrest and cell death [1,8]. In our laboratory, we have recently developed a method for quantitation of lung tissue depurination in intranasally ricin-intoxicated mice, and assessed the toxin’s effect on different pulmonary cell populations. Using this approach, we have demonstrated that intranasal ricin intoxication leads to massive depurination-derived damage of the pulmonary epithelium cells [9], which play a key role in maintaining the integrity and function of the respiratory system [10]. 

In addition to depurination-induced cell death and tissue damage, pulmonary ricin exposure results in a severe localized inflammation, associated with an intrapulmonary cytokine storm, massive neutrophil recruitment, and pulmonary edema, subsequently resulting in respiratory failure and death [3,11,12]. The clinical manifestation of ricin-induced respiratory damage in swine complies with the accepted diagnostic criteria for acute respiratory distress syndrome (ARDS) [13]. 

Deciphering the relative contribution of each of these processes, cell damage stemming directly from depurination, versus acute severe inflammation, to the pathology and prognosis of pulmonary ricin intoxication, is of considerable importance and is relevant for anti-ricin treatment development. To this end, we focused in the present study on the relative contribution of the inflammatory process to pulmonary ricinosis.

We have previously demonstrated that co-administration of the anti-inflammatory drugs doxycycline or ciprofloxacin with anti-ricin antibodies to mice intranasally exposed to lethal doses of ricin, significantly attenuated lung injury and improved treatment outcome in comparison to mice treated with anti-ricin antibodies alone. This improved protection was attributed to a significant quantitative reduction of various inflammatory related markers—cytokine response, neutrophil infiltration, and vascular hyperpermeability [11,14]. The anti-inflammatory effects of doxycycline or ciprofloxacin were significant, yet limited, and their application without co-administration of anti-ricin antibody did not confer protection. Furthermore, unlike doxycycline and ciprofloxacin, dexamethasone—a classical, potent first-line anti-inflammatory drug—was incapable of conferring protection when co-administered with anti-ricin antibody at 24 h post exposure [11]. This atypical anti-inflammatory response, as well as the inability of a drug-alone treatment to confer protection, prompted us to search for a non-pharmacologic anti-inflammatory tool in order to systematically analyze the contribution of the inflammatory process to the morbidity associated with pulmonary ricinosis.

Charting the progression of pulmonary ricin-induced toxicity in neutrophil-depleted mice may serve to define the role of inflammation in pulmonary ricinosis. As previously demonstrated, extensive time-evolved neutrophil infiltration takes place following pulmonary ricin exposure [15,16,17]. Neutrophils have a crucial role in the development and progression of both sterile [18,19,20] and infectious [21,22,23] lung injuries. These polymorphonuclear cells actively contribute to oxidative and proteolytic damages via secretion of reactive oxygen species, metalloproteinases, as well as other damage mediating factors [24,25]. Neutrophil depletion was shown to mitigate several forms of acute lung injury [18,21], and vice versa—effective manipulations/treatments that reduced lung injury parameters were shown to correlate with reduced pulmonary neutrophil counts [26]. The meager response to dexamethasone in pulmonary ricinosis previously mentioned, may also be relevant to the contribution of neutrophils to ricin-induced morbidity and mortality, since inflammation-activated neutrophils synthesize functionally inactive beta isoforms of the glucocorticoid receptor, rendering them less corticosteroid-sensitive [27]. Moreover, neutrophils are relatively insensitive to corticosteroid-mediated apoptosis [28,29]. 

In the present study, total body irradiation (TBI) served as a tool to prevent neutrophil recruitment to the lungs. TBI is a highly efficient immunosuppressive tool, extensively used in the research of bone marrow- [30,31] and hematopoietic stem cell- [32] transplantations, as well as in myelosuppression research [33] in mice. Generally, TBI induces not only neutropenia but also leukopenia, namely total white cell count depletion, and in some cases also erythrocyte/platelet depletion [33]. However, in our system, in which ricin in itself reduces T and B lymphocytes and NK cells while in parallel brings about a significant increase in neutrophil counts in lungs—TBI, which attenuates neutrophil migration to the lungs—could serve as a useful tool for elucidating their contribution to the pathology associated with ricin intoxication. 

Here we show that TBI-induced leukopenia/neutropenia considerably increased the mean time to death and alleviated the inflammatory response of mice following intranasal ricin intoxication, thereby expanding the therapeutic time window for anti-ricin antibody-based treatment in a significant manner.

## 2. Results

### 2.1. Effects of TBI on Peripheral Blood Count and Pulmonary Hematopoietic Cell Count Following Intranasal Ricin Intoxication

**Figure 1:**
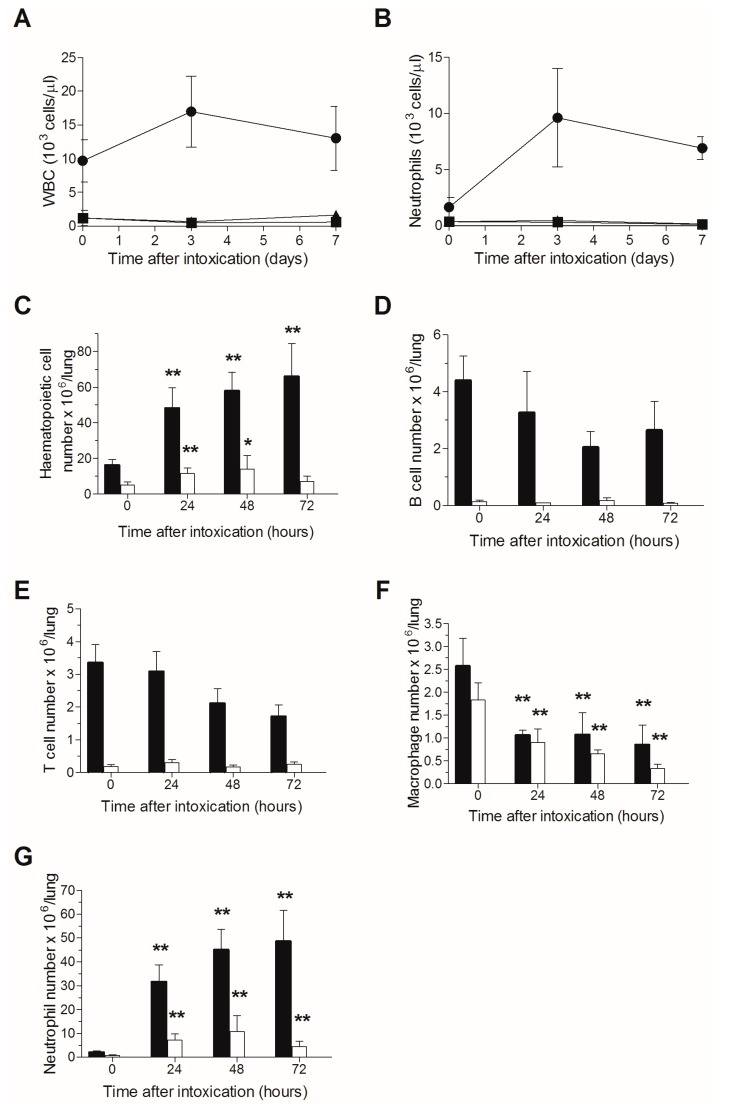
TBI-induced leukopenia in ricin intoxicated mice: Blood counts in mice following ricin intoxication. Naive or irradiated mice were intranasally intoxicated with ricin (circles and triangles, respectively), blood samples were withdrawn at the indicated time points and WBCs (**A**) and neutrophils (**B**) were quantified. Irradiated non-intoxicated mice served as control (squares). Irradiated (white bars) and non-irradiated (black bars) mice were intoxicated with ricin, lungs were harvested at different time-points following intoxication, and cells were quantified by flow cytometric analysis. Hematopoietic cells (**C**); B lymphocytes (**D**); T lymphocytes (**E**); macrophages (**F**); neutrophils (**G**). Number of animals per experimental group: A. *n* = 7–8 mice per group for all ricin-intoxicated mice; *n* = 2 mice per group for irradiated mice that were not intoxicated. B. *n* = 3 (irradiated) or 10 (non-irradiated) mice per group.* *p* < 0.05 in comparison to non-intoxicated mice; ** *p* < 0.01 in comparison to non-intoxicated mice.

A single sublethal irradiation dose of 6.5 Gy was previously reported to induce a stable severe leukopenia in naive CD-1 mice for at least 14 days, the nadir (minimal white blood cell count) observed at 3–9 days following irradiation, after which, an increase in white blood count was demonstrated between 9–14 days post-TBI, while full recovery was reached at 3–4 weeks following TBI [34]. Accordingly, in order to attain a stable neutropenia following ricin exposure, mice were subjected to TBI at a single dose of 6.5 Gy and were intranasally challenged three days later with a lethal dose of ricin. Peripheral blood leukopenia (Figure 1A), and specifically neutropenia (Figure 1B), were found to be stable in all mice subjected to TBI, whether exposed to ricin or not, until day 7 post ricin-exposure (at this time point, most of the ricin-intoxicated mice have died) in contrast to the prominent leukocytosis and neutrophilia observed in the non-irradiated ricin-intoxicated group. Quantitative analysis of pulmonary cells in non-irradiated mice revealed a time-dependent increase in hematopoietic cell counts from 10 to 70 × 10^6^ cells/lungs during the 72 h following intranasal ricin intoxication, while in irradiated ricin-intoxicated mice, the pulmonary hematopoietic count did not alter significantly during this period of time (5–10 × 10^6^ cells/lungs at all examined time points) and values were lower than those measured in naive non-irradiated mice (Figure 1C). 

FACS analysis of pulmonary hematopoietic cells demonstrated that following ricin-intoxication of non-irradiated mice, lymphocyte counts (T and B) were slightly reduced (Figure 1D,E), macrophage counts were reduced to a greater extent (Figure 1F), whereas neutrophil counts were extensively amplified (Figure 1G), all in line with previous observations at our laboratory [35]. In contrast, in irradiated mice, where the lymphocyte counts were dramatically reduced (Figure 1D,E), lymphocytopenia remained stable at all time points examined following ricin-intoxication and most importantly, neutrophil recruitment to the lungs was virtually abolished. Thus, at 72 h post-exposure, the pulmonary neutrophil count was 5 × 10^6^ cells/lungs in irradiated mice, as opposed to 5 × 10^7^ neutrophils/lungs in non-irradiated mice (Figure 1G). These results clearly indicate stable neutropenia following ricin intoxication of irradiated mice.

### 2.2. Lack of Additive Toxicity Following TBI in Ricin Intoxicated Mice

**Figure 2:**
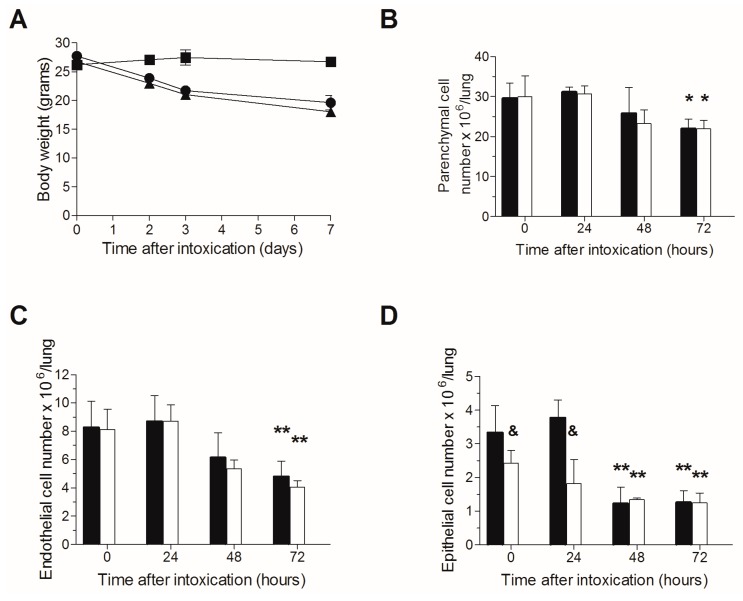
Lack of additive toxicity following TBI in ricin intoxicated mice: (**A**) Non-irradiated or irradiated mice (circles and triangles, respectively) were intranasally intoxicated with ricin, and body weights were determined at the indicated time points. Irradiated-non-intoxicated-mice served as control (squares). Irradiated (white bars) or non-irradiated (black bars) mice were intoxicated with ricin, lungs were harvested at the indicated time points and single cell suspensions were subjected to flow cytometric analysis for cell quantification; parenchymal cells (**B**); endothelial (**C**); and epithelial (**D**) cells. Number of animals per experimental group: A. *n* = 2 mice per group. B. *n* = 3 (irradiated) or 10 (non-irradiated) mice per group. * *p* < 0.05 in comparison to non-intoxicated mice; ** *p* < 0.01 in comparison to non-intoxicated mice; & *p* < 0.05 in comparison to non-irradiated mice at the same time point.

Since TBI per se may induce pulmonary damage, we next ruled out the possibility that excessive additive toxicity occurs following ricin intoxication of irradiated mice. Naive or irradiated mice were intranasally intoxicated with ricin (three days following TBI in the relevant group), body weights were monitored, and in addition, lungs were harvested at 0–72 h post exposure for cell quantification. Following intranasal ricin intoxication, 25–30% reductions in body weights were observed in both irradiated or naive mice subjected to ricin intoxication, without any noticeable difference between the two groups (Figure 2A). Likewise, parenchymal cell counts were similar in both irradiated and non-irradiated mice (Figure 2B), and a significant decrease in both endothelial (Figure 2C) and epithelial (Figure 2D) cells was displayed at 48 and 72 h post exposure, however, without significant additive cytotoxicity in the irradiated mice group. The epithelial cell count in the mice group subjected to TBI was somewhat lower than that measured for the non-irradiated mice before intoxication and at 24 h following intoxication, apparently due to the increased sensitivity of these cells to irradiation [36,37,38,39,40,41]. Overall, these results indicate for the lack of overt additive toxicity due to both irradiation and ricin intoxication, in comparison to ricin intoxication alone, enabling evaluation of the effect of neutrophil depletion in pulmonary ricinosis. 

### 2.3. TBI-Induced Extension of the Mean Time to Death in Ricin Intoxicated Mice

**Figure 3:**
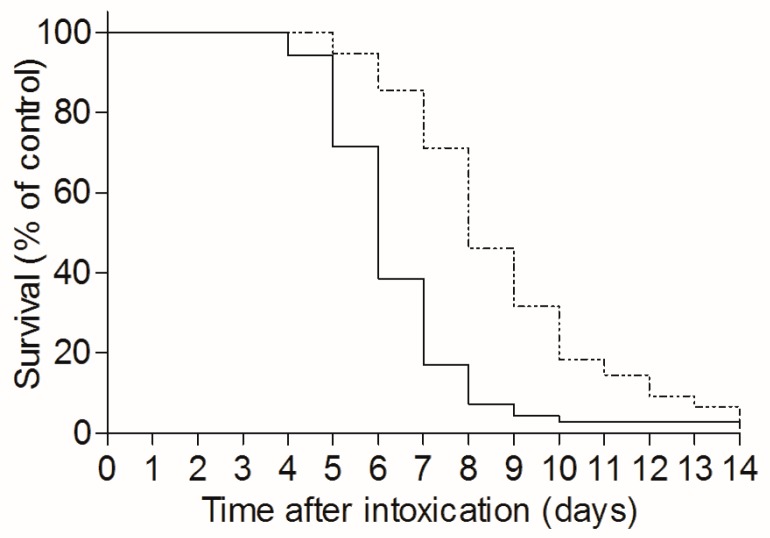
TBI-induced leukopenia extends the mean time to death in ricin intoxicated mice: irradiated (dashed line, *n* = 76) or naive mice (solid line, *n* = 70) were intranasally intoxicated with ricin (7 µg/kg). Mice survival was monitored until day 14 following intoxication.

To determine the effect of TBI on the time-course of pulmonary ricin-intoxication, naive and TBI-mice were intranasally intoxicated with ricin three days after irradiation, and the mean time to death (MTTD) of the two mice groups was monitored over a 14 day period. As seen, a significant increase in MTTD, from 6.3 days in non-irradiated mice to 8.7 days in the TBI group, was achieved (Figure 3).

As mentioned above [34], leukopenia is firmly stable in CD-1 mice during the nine days following irradiation, a gradual and moderate recovery of neutrophils is observed during the next four days, whereas between 14–28 days post-TBI, neutrophil counts increase gradually until full regeneration is achieved. The significant increase in MTTD of ricin-intoxicated mice described above, occurred when irradiation preceded intoxication by three days. To determine whether recovery of neutrophil production in irradiated mice reverses the apparent effect of TBI on MTTD, we extended our study to include groups of mice exposed to a lethal dose of ricin at later time points after irradiation, 9 days or 18 days post-TBI. Although the mice of both irradiated groups were similarly neutropenic at the time of intoxication, subsequent exposure to ricin caused a sharp increase in peripheral blood neutrophil counts, from 0.35 to 1.36 × 10^3^ cells/µL, only in the mice intoxicated at the latest time point, 18 days following TBI (Table 1). Furthermore, the MTTD value calculated for the mice exposed to ricin at 18 days post-TBI, 5.6 days, was markedly lower than the MTTD exhibited by mice exposed to ricin at 3 or 9 days post-TBI (8.1 and 8.4 days, respectively) and was similar to the MTTD value determined for non-irradiated ricin-intoxicated mice (6.3 days). These two parallel observations, the ability of bone marrow to produce neutrophils in mice exposed to ricin at day 18 post-TBI and the markedly lower MTTD exhibited by these mice following intoxication, suggests a causal linkage between the renewal of peripheral neutrophilia and the cancellation of TBI-induced MTTD prolongation. It should be mentioned that counts of leukocytes other than neutrophils, following intoxication at day 9 and 18 post irradiation were similar (data not shown), supporting our hypothesis that the noted changes in MTTD values following TBI are governed mainly by the presence or absence of neutrophils.

### 2.4. Anti-Inflammatory Effects Induced by TBI Following Intranasal Ricin Intoxication

To further characterize the effects of TBI on pulmonary ricinosis, we compared the occurrence and progression of various ricin-intoxication-associated inflammatory-related processes in irradiated and non-irradiated mice. To this end, bronchoalveolar lavage fluids (BALFs) were collected before and at 72 h post-intoxication, and pro-inflammatory cytokines, as well as edema and tissue degradation markers were measured. The pro-inflammatory cytokines examined were TNFα, IL-1β, and IL-6, which were previously determined to be elevated in mice lungs following ricin exposure [11,16]. The edema markers measured comprise total protein, as well as cholinesterase (ChE), the latter being a serum-resident enzyme which is normally confined to the bloodstream and appears at elevated levels in the lungs only in the case where the pulmonary epithelial–endothelial barrier was disrupted [11]. The tissue degradation markers measured included secretory phospholipase A2 (sPLA_2_), and xanthine oxidase (XO), representing lipolytic and oxidant activities, respectively. TNFα levels were found to be similarly elevated at 72 h post exposure in both irradiated and non-irradiated mice. In contrast, IL-1β levels in the non-irradiated and irradiated groups were 100 and 5 pg/mL, respectively, and IL-6 levels were 1700 and 360 pg/mL (~80% reduction), respectively, indicative of a reduced inflammatory response [11]. Likewise, significant attenuation of damage markers was noted in the irradiated mice. In the case of edema markers, approximately 50% reductions were determined in the irradiated mice (130 versus 270 mU/mL ChE; and 3 versus 5 mg/mL protein, in the BALFs of irradiated and not irradiated, ricin-intoxicated mice, respectively). The oxidative stress marker XO was also found to be lower, by nearly half, in the irradiated mice (2.5 and 4 mU/mL in TBI and non-irradiated groups, respectively). Thus, while a three-fold increase in sPLA_2_ was measured at 72 h after exposure to ricin in the BALFs of non-irradiated mice, the levels of this marker were very low already at the time of intoxication in the BALFs of the irradiated mice, and remained so until 72 h post exposure (Table 2). Taken together, these findings suggest that TBI-induced neutrophil depletion leads to a wide-range of anti-inflammatory effects following ricin intoxication.

To further probe the immunomodulatory effects of TBI, we next examined whether the irradiation allows extension of the therapeutic time window for anti-ricin antibody treatment of mice following ricin intoxication. To this end, non-irradiated and irradiated mice intranasally intoxicated with ricin (three days following TBI in the latter group), were treated with anti-ricin antibody at 48 or 72 h following intoxication, and survival rates were monitored over a period of 14 days. When antibody treatment was applied at 48 h post-exposure, survival rates of the irradiated mice were approximately 40%, while only negligible survival levels (4%) were documented for the non-irradiated mice treated with anti-ricin antibodies at this time point. Significant surviving rates (36%) were observed for irradiated mice treated at 72 h post exposure as well. At this late time point, antibody treatment offered no protection to the non-irradiated ricin-intoxicated mice. Interestingly, the irradiated mice exhibited similarly prolonged MTTD values in comparison to non-irradiated mice (approximately two days longer), whether or not they were subjected to antibody treatment (Table 3).

**Figure 4:**
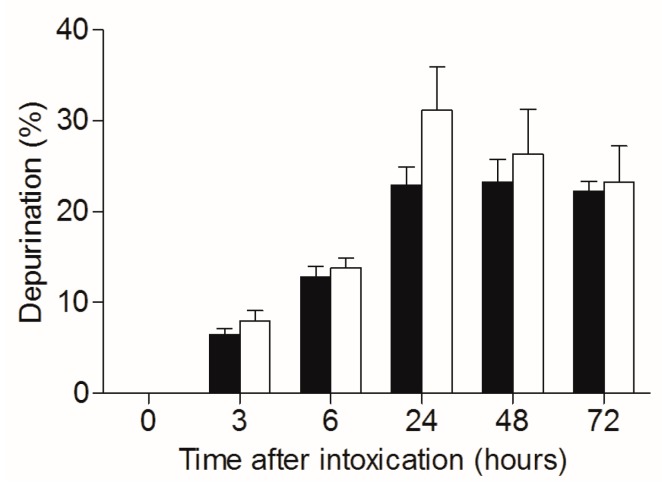
Catalytic damage of 28S rRNA in lungs of irradiated and non-irradiated mice intoxicated with ricin. Irradiated (white bars) and non-irradiated (black bars) mice were intranasally intoxicated with ricin (7 µg/kg), lungs were harvested at different time points following intoxication and catalytic damage (depurination) was quantified. *n* = 4.

As mentioned, ricin-related pathology could be attributed to both direct depurination-mediated cell damage and death, and on the other hand, to the indirect damage stemming from the raging inflammatory process, which in turn leads to respiratory insufficiency. The TBI-related dampening of ricin toxicity detailed above seems to be associated to with the mitigation of the inflammatory process. To evaluate whether irradiation of mice affects the catalytic performance of the toxin, irradiated and non-irradiated mice were intoxicated with ricin, lungs were harvested, and depurination ratios in the lung cells were calculated (Figure 4). Nearly equal depurination levels were observed for irradiated and non-irradiated mice at all time points examined, clearly demonstrating that ribosome depurination was not affected by the irradiation. Thus, one may conclude that the TBI-related beneficial effects observed in context of pulmonary ricinosis were entirely of an anti-inflammatory nature. 

## 3. Discussion

Pulmonary ricin intoxication is characterized by a confined lung pathology associated with cytokine storm, massive neutrophil infiltration, and ultimately, severe edema formation, leading to respiratory failure and death. Neutrophils are considered a major hallmark of ricin- [11,14,16], as well as non-ricin- [19,20,22,23] mediated lung injuries, where aggressive or prolonged neutrophil responses result in deleterious inflammatory conditions and tissue destruction, while on the other hand, decreased pulmonary neutrophil infiltration is associated with attenuation of injury severity [18,21,26]. 

In this work, TBI was employed to achieve a stable, long lasting neutropenia, aiming at selectively attenuating the inflammatory-related processes following ricin intoxication, without affecting the ribosomal damage induced by the catalytic activity of the toxin (28S rRNA depurination). Since pulmonary exposure to ricin induces neutrophil activation and recruitment, we first verified that irradiation intensity of 6.5 Gy, previously reported to induce a stable neutropenia in CD-1 mice [34], promotes stable neutropenia even when mice are subjected to intranasal ricin intoxication. Indeed, leukopenia/neutropenia remained stable in irradiated ricin-intoxicated mice at least for a week in the peripheral blood. The TBI-induced leukopenia was characterized by substantial reductions in pulmonary T- and B-lymphocyte counts (Figure 1D,E), and most notably in neutrophil counts. Although both pulmonary lymphocytopenia and neutropenia were observed at all time points tested following intoxication (Figure 1G), the TBI-induced effect is mainly related to the neutrophil depletion, since lymphocyte count decrease occurs following ricin intoxication regardless of irradiation, whereas neutrophil counts increase extensively following extravasation into the lungs, in non-irradiated mice intranasally exposed to ricin. It should be noted in this context, that while most hematopoietic-derived inflammatory cells are highly susceptible to ricin-induced protein synthesis arrest and cell death, ricin binds poorly if at all to neutrophils and hence, this cell type is not directly affected by exposure to the toxin [35].

Since irradiation in itself may injure various organs, including the lungs [36], we determined that under the experimental conditions of the present study, TBI did not inflict any notable damage to the ricin-intoxicated mice. Indeed, as reflected by the similar patterns of weight loss (Figure 2A) and parenchymal cell injury (Figure 2B) in both irradiated and non-irradiated mice, the stable leukopenia obtained by irradiation seems to be without overt toxicity. The single TBI-related deleterious effect noticed was a minor reduction in epithelial cell counts, in comparison to the non-irradiated mice (Figure 2C), whereas no observed TBI-mediated endothelial cell injury was noticed (Figure 2D).

While TBI did not improve survival rates following ricin intoxication, a significant increase in MTTD, from 6.3 to 8.7 days, was measured in ricin intoxicated mice subjected to TBI (Figure 3). This prolongation in time to death required a stable TBI-induced neutropenia, as MTTD prolongation was reversed when intoxication conditions allowed for peripheral blood neutrophilia formation in ricin intoxicated mice. Thus, prolonged MTTDs were measured in irradiated mice that were intoxicated at three and nine days following TBI, in which case peripheral neutropenia remained stable at three days following intoxication. In contrast, when mice were intoxicated at 18 days following TBI, a marked neutrophilia was exhibited at three days following intoxication, and indeed these mice displayed a considerably lower MTTD value, similar to that demonstrated by non-irradiated ricin-intoxicated mice (Table 1). Hence, it seems that the regeneration of neutrophil production capability by the bone marrow is critical for the development of full-blown pulmonary ricinosis.

In addition to neutrophil depletion, our findings clearly illustrate that TBI induces pronounced anti-inflammatory effects in ricin intoxicated mice (Table 2), as reflected by reduced cytokine response and damage markers in the BALFs collected at 72 h post exposure. IL-6, a prognostic biomarker of acute lung injury severity in pulmonary ricinosis [11] as well as in pulmonary abrinosis [42], was dramatically reduced by ~80%, in irradiated mice intranasally exposed to ricin, compared to the non-irradiated corresponding group. In addition, IL-1β levels of irradiated mice returned to the basal levels (namely the levels measured in naive mice) at 72 h after ricin intoxication, while significantly higher levels were measured in the ricin intoxicated, non-irradiated mice at this time point. It was previously demonstrated that IL-1β production is critical for the lung injury progression in pulmonary ricinosis, as markedly attenuated lung injury severity was observed in IL-1β depleted mice following intratracheal ricin exposure [16]. TNFα plays a critical role in lung pathologies, including ARDS [43]. However, the levels detected in the irradiated mice were similar to those measured in the non-irradiated mice following intoxication. TNFα is an early cytokine secreted primarily by activated monocytes or macrophages, which were shown to be strongly and rapidly affected by direct ricin activity [9,35] and therefore secretion of TNFα, which is not related to neutrophil activity, is not affected by irradiation. Neutrophils play a crucial role in the development of pulmonary vascular hyperpermeability [22,44]. Accordingly, edema markers were assessed in irradiated mice following intranasal ricin exposure. In comparison to non-irradiated ricin-intoxicated mice, ricin-intoxicated TBI-mice exhibited markedly reduced ChE and total protein levels (~40% reduction) in the BALF. Likewise, XO levels in BALFs of ricin-intoxicated mice were found significantly lower following irradiation. XO is an important source of reactive oxygen species (ROS) in lung pathologies [45,46]. In particular, XO was shown to actively contribute to pulmonary edema formation following ischemia/reperfusion (I/R), via direct ROS-induced pulmonary endothelium injury [47], as well as following LPS administration [48]. Levels of the lipolytic enzyme sPLA_2_, a potent mediator of inflammation responsible for hydrolysis and degradation of surfactant phospholipids [49,50], rose significantly after ricin intoxication only in non-irradiated mice. In contrast, its levels in the irradiated mice, which were lower than the levels measured in naive mice, did not alter following ricin intoxication at all time points examined. The markedly low levels of sPLA_2_ observed in irradiated mice are presumed to be a direct outcome of the ensuing leukopenia formation, since this enzyme is secreted mainly by the white blood cells macrophages, lymphocytes, NK cells, and neutrophils [50,51,52,53,54]. 

According to the literature, lung neutrophil infiltration is induced by IL-1β [16], IL-6 [55,56,57], XO [58,59,60], and sPLA_2_ [61,62]. The finding that these markers are significantly reduced in neutrophil-depleted mice intoxicated with ricin, may suggest that neutrophils operate upstream to these markers in a positive feedback loop, enhancing damage propagation in lung injuries, rather than being downstream responders to pro-inflammatory signals. 

Previously, we have shown that immunomodulators attenuate the inflammatory response and improve survival rates when co-administered to ricin-intoxicated mice with anti-ricin antibody. The results obtained in this work provide supplementary data regarding the clinical importance of inflammatory mitigation in the course of pulmonary ricinosis, since TBI expands the therapeutic window in irradiated animals treated with anti-ricin antibody. While it was impossible to rescue non-irradiated mice by anti-ricin antibody treatment at 48 h post exposure, the survival levels of TBI-subjected mice were as high as ~40% at this point of care. Moreover, treating irradiated mice as late as 72 h following intoxication resulted in a similar survival rate, attesting to the significant anti-inflammatory effect of TBI (Table 3).

We are fully aware that in addition to neutropenia, TBI induced stable lymphocytopenia and in our system the role of these cells could not be excluded. However, the prolongation of MTTD was shown to be dependent on neutrophil production, and not T- and B-cell production. We would like to point out in this context that since this study was performed with outbred mice, employing an adoptive transfer approach to prove unequivocally the role of the neutrophils in promotion of toxicity is not possible. Understandably, TBI is not an applicable clinical countermeasure against ricin intoxication, however, the results obtained in this work strongly strengthen our findings from the past, indicating that attenuation of inflammation is critical for optimal treatment of pulmonary ricinosis, especially if antibody-based treatment begins at late time points following intoxication [11,14]. Furthermore, the present study strongly suggests that clinical approaches aimed to attenuate neutrophil-mediated damage could be implemented in the future in the development of an effective anti-ricin medical countermeasure. Some drug candidates aiming at suppressing neutrophil activity are clinically available, and others are under pre-clinical evaluation [63]. Moreover, since TBI induces an immunosuppressive effect, immunosuppressant agents could also be tested as a treatment for pulmonary ricinosis, pending adverse reactions and toxicity. The present study shows that improved survival rates of ricin-intoxicated mice could be reached only following irradiation in conjunction with anti-ricin antibody treatment. The fact that survival rates were not increased by attenuation of inflammation via irradiation in itself seems to stem on one hand from the short term effect of irradiation and on the other hand, from the direct non-inflammogenic effects of ricin, namely depurination induced tissue destruction, which is not affected by TBI.

## 4. Materials and Methods

### 4.1. Ricin Preparation

Crude ricin was prepared from seeds of endemic *Ricinus communis*, essentially as described before [64]. Briefly, seeds were homogenized in a blender (Waring, Torrington, CT, USA) in 5% acetic acid (Merck, Darmstadt, Germany)/phosphate buffer (Na_2_HPO_4_, pH 7.4, Sigma-Aldrich, Rehovot, Israel). The homogenate was centrifuged and the clarified supernatant containing the toxin was subjected to ammonium sulfate (Merck, Darmstadt, Germany) precipitation (60% saturation). The precipitate was dissolved in PBS (Bioligical Industries, Beit Haemek, Israel) and dialyzed extensively against the same buffer. The toxin preparation appeared on a Coomassie Blue (Bio-Rad, Rishon Le Zion, Israel) stained non-reducing 10% polyacrylamide gel (ThermoFisher Scientific, Carlsbad, CA, USA) as two major bands of molecular weight of approximately 65 kDa (=ricin toxin, ~80%) and 120 kDa (=*ricinus communis* agglutinin, RCA, ~20%). Protein concentration was determined as 2.86 mg/mL by 280 nm absorption (NanoDrop 2000, Thermo Fisher Scientific, Waltham, MA, USA). 

### 4.2. Anti-Ricin Antibodies

Rabbit polyclonal anti-ricin antibodies were prepared as described before [11]. 

### 4.3. Animal Studies

Animal experiments were performed in accordance with the Israeli law and were approved by the Ethics Committee for animal experiments at the Israel Institute for Biological Research (project identification code M-01-2012, date of approval 2 January 2012). Treatment of animals was in accordance with regulations outlined in the USDA Animal Welfare Act and the conditions specified in the National Institute of Health Guide for Care and Use of Laboratory Animals. 

All animals in this study were female CD-1 mice (Charles River Laboratories Ltd., Margate, UK) weighing 27–32 grams. Prior to exposure, animals were habituated to the experimental animal unit for five days. All mice were housed in filter-top cages in an environmentally controlled room and maintained at 21 ± 2 °C and 55 ± 10% humidity. Lighting was set to mimic a 12/12 h dawn to dusk cycle. Animals had access to food and water ad libitum.

For intoxication, mice were anesthetized by an intraperitoneal injection of ketamine (1.9 mg/mouse, Vetoquinol, Lure, France) and xylazine (0.19 mg/mouse, Eurovet Animal Health, AD Bladel, The Netherlands). Crude ricin (50 μL; 7 μg/kg diluted in PBS) was applied intranasally (2 × 25 μL) and mortality was monitored over 14 days. Preceding these studies, we determined that 3.5 μg crude ricin/kg body weight is approximately equivalent to one mouse (intranasal) LD_50_ (95% confidence intervals of 2.3 to 4.5 μg/kg body weight). 

A volume of 100 µL of anti-ricin antibody preparation was delivered intravenously at the indicated times following intoxication.

### 4.4. Total Body Irradiation (TBI) Protocol

Mice were subjected to a single dose of 6.5 Gy whole body irradiation. For this purpose, the X-ray biological irradiator XRAD 320 (Precision X-ray, North Branford, CT, USA) at the Weizmann Institute of Science was used. The irradiation time was approximately 520 s.

### 4.5. Peripheral Blood Counts

White blood cells- and neutrophil-counts were determined in peripheral blood. Samples at a volume of 50 μL were collected from the tail vein of mice into EDTA containing tubes (BD, Franklin Lakes, NJ, USA) and were analyzed using Veterinary Multi-species Hematology System Hemavet 850 (Drew Scientific, Miami Lakes, FL, USA).

### 4.6. Flow Cytometry of the Lungs

Lungs were harvested, cut into small pieces, and digested for 2 h at 37 °C with 4 mg/mL collagenase D (Roche, Mannheim, Germany) in PBS Ca^+2^ Mg^+2^ (Biological Industries, Beit Haemek, Israel). The tissue was then meshed through a 40 μm cell strainer and red blood cells were lysed. Cells were co-stained for surface markers in a flow cytometry buffer (PBS with 2% FCS, 0.1% sodium azide, and 5 mM EDTA) as previously described [35] and analyzed using FACSCalibur (BD Biosciences, San Jose, CA, USA) and FlowJo software (version 7.1.2, Tree Star, Ashland, OR, USA, 2007).

### 4.7. Bronchoalveolar Lavage Fluid (BALF) Preparation and Analysis

BALFs, collected by instillation of 1 mL PBS at room temperature, were centrifuged at 3000 rpm at 4 °C for 10 min. Supernatants were collected and stored at −20 °C until further use.

BALF levels of TNFα, IL-1β, and IL-6 were determined by ELISA (R&D systems, Minneapolis, MN, USA). 

Cholinesterase (ChE) enzymatic activity was measured according to Ellman [65]. Assays were performed in the presence of 0.5 mM acetylthiocholine (Sigma-Aldrich, Rehovot, Israel), 50 mM sodium phosphate buffer pH 8.0 (Sigma-Aldrich, Rehovot, Israel), 0.1 mg/mL BSA (Sigma-Aldrich, Rehovot, Israel), and 0.3 mM 5,5′-dithiobis-(2-nitrobenzoic acid, Sigma-Aldrich, Rehovot, Israel). The assay was carried out at 27 °C and monitored by a Thermomax microplate reader (Molecular Devices, Ramsey, MN, USA). Protein levels in BALF were determined by 280 nm absorption (NanoDrop 2000, ThermoFisher Scientific, Waltham, MA, USA). Xanthine oxidase (XO, Molecular Probes, Eugene, OR, USA) in BALF was determined by an activity assay kit. 

Levels of secretory phospholipase A2 (sPLA_2_) were determined by an activity assay kit (Assay Designs, Ann Arbor, MI, USA).

### 4.8. Depurination Assay

Depurination was quantified as previously described [9]. Briefly, reverse transcriptase (RT) reaction was conducted with two oligonucleotids primers: the first, R-1-GGTAGACACCCTAATACT marked with FAM, and the second, R-HEX-CTTTGATTGGTCCTAAGGGAGTCATT marked with HEX. The primers and RNA were incubated with a RT mix containing M-MLV RT, DTT, dNTPs, and RNasin (Promega, Madison, WI, USA), for 20 min incubation at 37 °C and then another 20 min at 48 °C. The cDNA that was produced in the reaction was later separated by electrophoresis in the GeneScan (GeneScan, ABI PRISM 310 Genetic Analyzer, Applied Biosystems, Thermo Fisher Scientific, Waltham, MA, USA). ROX (MapMarker 400, BioVentures, Wellesley, MA, USA) served as a size marker.

### 4.9. Statistical Analysis

Individual groups were compared using unpaired *t*-test analysis. To estimate *p* values, all statistical analyses were interpreted in a two-tailed manner. Values of *p* < 0.05 were considered to be statistically significant. Kaplan–Meier analysis was performed for survival curves. All data is presented as means ± SEM.

## Figures and Tables

**Table 1:** TBI-induced stable leukopenia extends the mean time to death in ricin intoxicated mice. Mice were intoxicated at the indicated time-points following irradiation (Intoxication Time). Blood neutrophil counts were determined prior to intoxication and three days following intoxication. MTTD was determined. * *p* < 0.05 in comparison to neutrophil counts at intoxication day. ** *p* < 0.01 in comparison to neutrophil counts at intoxication day.

Intoxication Time	Blood Neutrophil Count (10^3^ Cells/µL)		MTTD (Days)
	At Exposure Day	3 Days Post Exposure	
Non irradiated mice	1.53 ± 0.60 (*n* = 12)	13.43 ± 1.56 ** (*n* = 3)	6.3 (*n* = 18)
3 days post irradiation	0.18 ± 0.09 (*n* = 9)	0.40 ± 0.45 (*n* = 4)	8.1 (*n* = 11)
9 days post irradiation	0.29 ± 0.26 (*n* = 3)	0.50 ± 0.23 (*n* = 2)	8.4 (*n* = 14)
18 days post irradiation	0.35 ± 0.12 (*n* = 3)	11.36 ± 7.25 * (*n* = 4)	5.6 (*n* = 9)

**Table 2:** Pro-inflammatory markers in the BALFs of irradiated and non-irradiated mice following ricin intoxication. Irradiated (TBI) or non-irradiated (none) mice were intranasally intoxicated with ricin (7 µg/kg) and BALF samples collected before (0) or 72 h (72) after intoxication were monitored for TNFα, IL-1β, IL-6, cholinesterase (ChE), protein, xanthine oxidase (XO), and secretory phospholipase A2 (sPLA_2_). *n* = 3–5. * *p* < 0.05 between tested group and control (*t* = 0). ** *p* < 0.01 between tested group and control. ^#^ *p* < 0.05 in comparison to parallel tested group of non-irradiated mice at the same time point post exposure. ^##^ *p* < 0.01 in comparison to parallel tested group of non-irradiated mice at the same time point post exposure.

Marker	Pre-Intoxication Treatment	Time after Exposure (h)	
		0	72
TNFα (pg/mL)	none	37 ± 16	80 ± 2 7 *
	TBI	35 ± 5	106 ± 32 *
IL-1β (pg/mL)	none	0 ± 0	108 ± 76
	TBI	3 ± 4	4 ± 6 ^#^
IL-6 (pg/mL)	none	3 ± 2	1695 ± 711 **
	TBI	0 ± 0	363 ± 99 *^##^
ChE (mU/mL)	none	3 ± 1	273 ± 38 **
	TBI	5 ± 1	130 ± 38 **^##^
Protein (mg/mL)	none	0.4 ± 0.1	5.2 ± 1.1 **
	TBI	0.7 ± 0.1	2.8 ± 0.5 **^##^
XO (mU/mL)	none	0.3 ± 0.3	3.7 ± 0.6 **
	TBI	0.2 ± 0.1	2.4 ± 0.6 **^##^
sPLA_2_ (U/mL)	none	7.8 ± 5.8	24.5 ± 8.7 *
	TBI	1.8 ± 1.8	1.6 ± 3.2 ^##^

**Table 3:** Survival rates and mean time to death values of irradiated and non-irradiated ricin-intoxicated mice subjected to post-exposure anti-ricin antibody treatment. Irradiated (TBI) or non-irradiated (none) mice were intranasally intoxicated with ricin (7 µg/kg) and then treated or not (---) with anti-ricin antibody at 48 or 72 h post exposure.

Anti-Ricin Ab Treatment Following i.n. Exposure (2LD_50_)						
Time of Ab Treatment (h)	---		48		72	
Pretreatment of Mice	none	TBI	none	TBI	none	TBI
% survival (survivors/total)	1 (1/70)	1 (1/76)	4 (1/23)	42 (10/24)	0 (0/7)	36 (4/11)
MTTD (days)	6.4	9.4	6.0	8.7	3.6	8.4

## References

[1] Olsnes S., Kozlov J.V. (2001). Ricin. Toxicon.

[2] Greenfield R.A., Brown B.R., Hutchins J.B., Iandolo J.J., Jackson R., Slater L.N., Bronze M.S. (2002). Microbiological, biological, and chemical weapons of warfare and terrorism. Am. J. Med. Sci..

[3] Audi J., Belson M., Patel M., Schier J., Osterloh J. (2005). Ricin poisoning: A comprehensive review. JAMA.

[4] Endo Y., Tsurugi K. (1987). Rna n-glycosidase activity of ricin a-chain. Mechanism of action of the toxic lectin ricin on eukaryotic ribosomes. J. Biol. Chem..

[5] Hartley M.R., Lord J.M. (2004). Cytotoxic ribosome-inactivating lectins from plants. Biochim. Biophys. Acta.

[6] Olmo N., Turnay J., Gonzalez de Buitrago G., Lopez de Silanes I., Gavilanes J.G., Lizarbe M.A. (2001). Cytotoxic mechanism of the ribotoxin alpha-sarcin. Induction of cell death via apoptosis. Eur. J. Biochem..

[7] Olsnes S., Fernandez-Puentes C., Carrasco L., Vazquez D. (1975). Ribosome inactivation by the toxic lectins abrin and ricin. Kinetics of the enzymic activity of the toxin a-chains. Eur. J. Biochem..

[8] Olivieri F., Prasad V., Valbonesi P., Srivastava S., Ghosal-Chowdhury P., Barbieri L., Bolognesi A., Stirpe F. (1996). A systemic antiviral resistance-inducing protein isolated from clerodendrum inerme gaertn. Is a polynucleotide:Adenosine glycosidase (ribosome-inactivating protein). FEBS Lett..

[9] Falach R., Sapoznikov A., Gal Y., Israeli O., Leitner M., Seliger N., Ehrlich S., Kronman C., Sabo T. (2016). Quantitative profiling of the in vivo enzymatic activity of ricin reveals disparate depurination of different pulmonary cell types. Toxicol. Lett..

[10] Knight D.A., Holgate S.T. (2003). The airway epithelium: Structural and functional properties in health and disease. Respirology.

[11] Gal Y., Mazor O., Alcalay R., Seliger N., Aftalion M., Sapoznikov A., Falach R., Kronman C., Sabo T. (2014). Antibody/doxycycline combined therapy for pulmonary ricinosis: Attenuation of inflammation improved survival of ricin-intoxicated mice. Toxicol. Rep..

[12] Wilhelmsen C.L., Pitt M.L. (1996). Lesions of acute inhaled lethal ricin intoxication in rhesus monkeys. Vet. Pathol..

[13] Katalan S., Falach R., Rosner A., Goldvaser M., Brosh-Nissimov T., Dvir A., Mizrachi A., Goren O., Cohen B., Gal Y. (2017). A novel swine model of ricin-induced acute respiratory distress syndrome. Dis. Model. Mech..

[14] Gal Y., Sapoznikov A., Falach R., Ehrlich S., Aftalion M., Sabo T., Kronman C. (2016). Potent antiedematous and protective effects of ciprofloxacin in pulmonary ricinosis. Antimicrob. Agents Chemother..

[15] DaSilva L., Cote D., Roy C., Martinez M., Duniho S., Pitt M.L., Downey T., Dertzbaugh M. (2003). Pulmonary gene expression profiling of inhaled ricin. Toxicon.

[16] Lindauer M.L., Wong J., Iwakura Y., Magun B.E. (2009). Pulmonary inflammation triggered by ricin toxin requires macrophages and il-1 signaling. J. Immunol..

[17] Poli M.A., Rivera V.R., Pitt M.L., Vogel P. (1996). Aerosolized specific antibody protects mice from lung injury associated with aerosolized ricin exposure. Toxicon.

[18] Abraham E., Carmody A., Shenkar R., Arcaroli J. (2000). Neutrophils as early immunologic effectors in hemorrhage- or endotoxemia-induced acute lung injury. Am. J. Physiol. Lung Cell. Mol. Physiol..

[19] Till G.O., Johnson K.J., Kunkel R., Ward P.A. (1982). Intravascular activation of complement and acute lung injury. Dependency on neutrophils and toxic oxygen metabolites. J. Clin. Investig..

[20] Yan B., Chen F., Xu L., Xing J., Wang X. (2017). HMGB1-TLR4-IL23-IL17A axis promotes paraquat-induced acute lung injury by mediating neutrophil infiltration in mice. Sci. Rep..

[21] Gao X.P., Liu Q., Broman M., Predescu D., Frey R.S., Malik A.B. (2005). Inactivation of cd11b in a mouse transgenic model protects against sepsis-induced lung pmn infiltration and vascular injury. Physiol. Genom..

[22] Koma T., Yoshimatsu K., Nagata N., Sato Y., Shimizu K., Yasuda S.P., Amada T., Nishio S., Hasegawa H., Arikawa J. (2014). Neutrophil depletion suppresses pulmonary vascular hyperpermeability and occurrence of pulmonary edema caused by hantavirus infection in c.B-17 scid mice. J. Virol..

[23] Sercundes M.K., Ortolan L.S., Debone D., Soeiro-Pereira P.V., Gomes E., Aitken E.H., Neto A.C., Russo M., MR D.I.L., Alvarez J.M. (2016). Targeting neutrophils to prevent malaria-associated acute lung injury/acute respiratory distress syndrome in mice. PLoS Pathog..

[24] Segel G.B., Halterman M.W., Lichtman M.A. (2011). The paradox of the neutrophil’s role in tissue injury. J. Leukoc. Biol..

[25] Wright H.L., Moots R.J., Bucknall R.C., Edwards S.W. (2010). Neutrophil function in inflammation and inflammatory diseases. Rheumatology.

[26] Zhao Y., Sharma A.K., LaPar D.J., Kron I.L., Ailawadi G., Liu Y., Jones D.R., Laubach V.E., Lau C.L. (2011). Depletion of tissue plasminogen activator attenuates lung ischemia-reperfusion injury via inhibition of neutrophil extravasation. Am. J. Physiol. Lung Cell. Mol. Physiol..

[27] Strickland I., Kisich K., Hauk P.J., Vottero A., Chrousos G.P., Klemm D.J., Leung D.Y. (2001). High constitutive glucocorticoid receptor beta in human neutrophils enables them to reduce their spontaneous rate of cell death in response to corticosteroids. J. Exp. Med..

[28] Cox G. (1995). Glucocorticoid treatment inhibits apoptosis in human neutrophils. Separation of survival and activation outcomes. J. Immunol..

[29] Daffern P.J., Jagels M.A., Hugli T.E. (1999). Multiple epithelial cell-derived factors enhance neutrophil survival. Regulation by glucocorticoids and tumor necrosis factor-alpha. Am. J. Respir. Cell Mol. Biol..

[30] Cui Y.Z., Hisha H., Yang G.X., Fan T.X., Jin T., Li Q., Lian Z., Ikehara S. (2002). Optimal protocol for total body irradiation for allogeneic bone marrow transplantation in mice. Bone Marrow Transplant..

[31] Schwartz E., Lapidot T., Gozes D., Singer T.S., Reisner Y. (1987). Abrogation of bone marrow allograft resistance in mice by increased total body irradiation correlates with eradication of host clonable t cells and alloreactive cytotoxic precursors. J. Immunol..

[32] Shen H., Yu H., Liang P.H., Cheng H., XuFeng R., Yuan Y., Zhang P., Smith C.A., Cheng T. (2012). An acute negative bystander effect of gamma-irradiated recipients on transplanted hematopoietic stem cells. Blood.

[33] Neelis K.J., Visser T.P., Dimjati W., Thomas G.R., Fielder P.J., Bloedow D., Eaton D.L., Wagemaker G. (1998). A single dose of thrombopoietin shortly after myelosuppressive total body irradiation prevents pancytopenia in mice by promoting short-term multilineage spleen-repopulating cells at the transient expense of bone marrow-repopulating cells. Blood.

[34] Heissig B., Rafii S., Akiyama H., Ohki Y., Sato Y., Rafael T., Zhu Z., Hicklin D.J., Okumura K., Ogawa H. (2005). Low-dose irradiation promotes tissue revascularization through vegf release from mast cells and mmp-9-mediated progenitor cell mobilization. J. Exp. Med..

[35] Sapoznikov A., Falach R., Mazor O., Alcalay R., Gal Y., Seliger N., Sabo T., Kronman C. (2015). Diverse profiles of ricin-cell interactions in the lung following intranasal exposure to ricin. Toxins.

[36] Ghafoori P., Marks L.B., Vujaskovic Z., Kelsey C.R. (2008). Radiation-induced lung injury. Assessment, management, and prevention. Oncology.

[37] Marks L.B., Yu X., Vujaskovic Z., Small W., Folz R., Anscher M.S. (2003). Radiation-induced lung injury. Semin. Radiat. Oncol..

[38] Medhora M., Gao F., Jacobs E.R., Moulder J.E. (2012). Radiation damage to the lung: Mitigation by angiotensin-converting enzyme (ace) inhibitors. Respirology.

[39] Mehta V. (2005). Radiation pneumonitis and pulmonary fibrosis in non-small-cell lung cancer: Pulmonary function, prediction, and prevention. Int. J. Radiat. Oncol. Biol. Phys..

[40] Pan J., Su Y., Hou X., He H., Liu S., Wu J., Rao P. (2012). Protective effect of recombinant protein sod-tat on radiation-induced lung injury in mice. Life Sci..

[41] Tsoutsou P.G., Koukourakis M.I. (2006). Radiation pneumonitis and fibrosis: Mechanisms underlying its pathogenesis and implications for future research. Int. J. Radiat. Oncol. Biol. Phys..

[42] Sabo T., Gal Y., Elhanany E., Sapoznikov A., Falach R., Mazor O., Kronman C. (2015). Antibody treatment against pulmonary exposure to abrin confers significantly higher levels of protection than treatment against ricin intoxication. Toxicol. Lett..

[43] Mukhopadhyay S., Hoidal J.R., Mukherjee T.K. (2006). Role of tnfalpha in pulmonary pathophysiology. Respir. Res..

[44] Finsterbusch M., Voisin M.B., Beyrau M., Williams T.J., Nourshargh S. (2014). Neutrophils recruited by chemoattractants in vivo induce microvascular plasma protein leakage through secretion of tnf. J. Exp. Med..

[45] Komaki Y., Sugiura H., Koarai A., Tomaki M., Ogawa H., Akita T., Hattori T., Ichinose M. (2005). Cytokine-mediated xanthine oxidase upregulation in chronic obstructive pulmonary disease’s airways. Pulm. Pharmacol. Ther..

[46] Wright R.M., Ginger L.A., Kosila N., Elkins N.D., Essary B., McManaman J.L., Repine J.E. (2004). Mononuclear phagocyte xanthine oxidoreductase contributes to cytokine-induced acute lung injury. Am. J. Respir. Cell Mol. Biol..

[47] Grosso M.A., Brown J.M., Viders D.E., Mulvin D.W., Banerjee A., Velasco S.E., Repine J.E., Harken A.H. (1989). Xanthine oxidase-derived oxygen radicals induce pulmonary edema via direct endothelial cell injury. J. Surg. Res..

[48] Faggioni R., Gatti S., Demitri M.T., Delgado R., Echtenacher B., Gnocchi P., Heremans H., Ghezzi P. (1994). Role of xanthine oxidase and reactive oxygen intermediates in lps- and tnf-induced pulmonary edema. J. Lab. Clin. Med..

[49] Arbibe L., Koumanov K., Vial D., Rougeot C., Faure G., Havet N., Longacre S., Vargaftig B.B., Bereziat G., Voelker D.R. (1998). Generation of lyso-phospholipids from surfactant in acute lung injury is mediated by type-ii phospholipase a2 and inhibited by a direct surfactant protein a-phospholipase a2 protein interaction. J. Clin. Investig..

[50] Touqui L., Arbibe L. (1999). A role for phospholipase a2 in ards pathogenesis. Mol. Med. Today.

[51] Costa-Junior H.M., Hamaty F.C., da Silva Farias R., Einicker-Lamas M., da Silva M.H., Persechini P.M. (2006). Apoptosis-inducing factor of a cytotoxic t cell line: Involvement of a secretory phospholipase a2. Cell Tissue Res..

[52] Marshall L.A., Bolognese B., Roshak A. (1999). Respective roles of the 14 kda and 85 kda phospholipase a2 enzymes in human monocyte eicosanoid formation. Adv. Exp. Med. Biol..

[53] Rosenthal M.D., Gordon M.N., Buescher E.S., Slusser J.H., Harris L.K., Franson R.C. (1995). Human neutrophils store type ii 14-kda phospholipase a2 in granules and secrete active enzyme in response to soluble stimuli. Biochem. Biophys. Res. Commun..

[54] Von Allmen C.E., Schmitz N., Bauer M., Hinton H.J., Kurrer M.O., Buser R.B., Gwerder M., Muntwiler S., Sparwasser T., Beerli R.R. (2009). Secretory phospholipase a2-iid is an effector molecule of cd4+cd25+ regulatory t cells. Proc. Natl. Acad. Sci. USA.

[55] Jones M.R., Quinton L.J., Simms B.T., Lupa M.M., Kogan M.S., Mizgerd J.P. (2006). Roles of interleukin-6 in activation of stat proteins and recruitment of neutrophils during escherichia coli pneumonia. J. Infect. Dis..

[56] Leemans J.C., Vervoordeldonk M.J., Florquin S., van Kessel K.P., van der Poll T. (2002). Differential role of interleukin-6 in lung inflammation induced by lipoteichoic acid and peptidoglycan from staphylococcus aureus. Am. J. Respir. Crit. Care Med..

[57] Rijneveld A.W., van den Dobbelsteen G.P., Florquin S., Standiford T.J., Speelman P., van Alphen L., van der Poll T. (2002). Roles of interleukin-6 and macrophage inflammatory protein-2 in pneumolysin-induced lung inflammation in mice. J. Infect. Dis..

[58] Granell S., Gironella M., Bulbena O., Panes J., Mauri M., Sabater L., Aparisi L., Gelpi E., Closa D. (2003). Heparin mobilizes xanthine oxidase and induces lung inflammation in acute pancreatitis. Crit. Care Med..

[59] Nielsen V.G., Tan S., Weinbroum A., McCammon A.T., Samuelson P.N., Gelman S., Parks D.A. (1996). Lung injury after hepatoenteric ischemia-reperfusion: Role of xanthine oxidase. Am. J. Respir. Crit. Care Med..

[60] Terada L.S., Dormish J.J., Shanley P.F., Leff J.A., Anderson B.O., Repine J.E. (1992). Circulating xanthine oxidase mediates lung neutrophil sequestration after intestinal ischemia-reperfusion. Am. J. Physiol..

[61] Lee Y.M., Hybertson B.M., Terada L.S., Repine A.J., Cho H.G., Repine J.E. (1997). Mepacrine decreases lung leak in rats given interleukin-1 intratracheally. Am. J. Respir. Crit. Care Med..

[62] Munoz N.M., Meliton A.Y., Meliton L.N., Dudek S.M., Leff A.R. (2009). Secretory group v phospholipase a2 regulates acute lung injury and neutrophilic inflammation caused by lps in mice. Am. J. Physiol. Lung Cell. Mol. Physiol..

[63] Burgos R.A., Hidalgo M.A., Figueroa C.D., Conejeros I., Hancke J.L. (2009). New potential targets to modulate neutrophil function in inflammation. Mini Rev. Med. Chem..

[64] Lin J.Y., Liu S.Y. (1986). Studies on the antitumor lectins isolated from the seeds of ricinus communis (castor bean). Toxicon.

[65] Ellman G.L., Courtney K.D., Andres V., Feather-Stone R.M. (1961). A new and rapid colorimetric determination of acetylcholinesterase activity. Biochem. Pharmacol..

